# Emergency Department Overcrowding: Understanding the Factors to Find Corresponding Solutions

**DOI:** 10.3390/jpm12020279

**Published:** 2022-02-14

**Authors:** Gabriele Savioli, Iride Francesca Ceresa, Nicole Gri, Gaia Bavestrello Piccini, Yaroslava Longhitano, Christian Zanza, Andrea Piccioni, Ciro Esposito, Giovanni Ricevuti, Maria Antonietta Bressan

**Affiliations:** 1 Emergency Medicine and Surgery, IRCCS Fondazione Policlinico San Matteo, 27100 Pavia, Italy; gabrielesavioli@gmail.com (G.S.); mita.bressan@gmail.com (M.A.B.); 2 PhD School in Experimental Medicine, Department of Clinical-Surgical, Diagnostic and Pediatric Sciences, University of Pavia, 27100 Pavia, Italy; 3 Emergency Department, Ospedale Civile Vigevano, 27029 Vigevano, Italy; irideceresa@gmail.com; 4 Department of Internal Medicine and Therapeutics, University of Pavia, 27100 Pavia, Italy; nicole.gri01@universitadipavia.it (N.G.); gaia.bavestrellopic01@universitadipavia.it (G.B.P.); 5 School of Master in Emergency Medicine, Université Libre de Bruxelles, 1050 Brussels, Belgium; 6 Foundation “Ospedale Alba-Bra Onlus”, Department of Emergency Medicine, Anesthesia and Critical Care Medicine, Michele and Pietro Ferrero Hospital, 12060 Verduno, Italy; lon.yaro@gmail.com; 7 Research Training Innovation Infrastructure, Research and Innovation Department, Azienda Ospedaliera SS Antonio e Biagio e Cesare Arrigo, 15121 Alessandria, Italy; 8 Department of Emergency Medicine, Policlinico Agostino Gemelli, Catholic University of Sacred Heart, 00168 Rome, Italy; andrea.piccioni@policlinicogemelli.it; 9 Unit of Nephrology and Dialysis, ICS Maugeri, University of Pavia, 27100 Pavia, Italy; ciro.esposito@unipv.it; 10 School of Pharmacy, Department of Drug Sciences, University of Pavia, 27100 Pavia, Italy; giovanni.ricevuti@unipv.it

**Keywords:** overcrowding, emergency department, length of stay, waiting time, inpatient boarding, triage, hospital emergency services, ed patient flow, ambulance diversion, emergency outpatient unit, patient safety

## Abstract

It is certain and established that overcrowding represents one of the main problems that has been affecting global health and the functioning of the healthcare system in the last decades, and this is especially true for the emergency department (ED). Since 1980, overcrowding has been identified as one of the main factors limiting correct, timely, and efficient hospital care. The more recent COVID-19 pandemic contributed to the accentuation of this phenomenon, which was already well known and of international interest. Considering what would appear to be a trivial definition of overcrowding, it may seem simple for the reader to hypothesize solutions for what seems to be one of the most avoidable problems affecting the hospital system. However, proposing solutions to overcrowding, as well as their implementation, cannot be separated from a correct and precise definition of the issue, which must consider the main causes and aggravating factors. In light of the need of finding solutions that can put an end to hospital overcrowding, this review aims, through a review of the literature, to summarize the triggering factors, as well as the possible solutions that can be proposed.

## 1. Introduction

In order to proceed with a narrative analysis on overcrowding, it seems useful to provide some definitions, although defining and quantifying overcrowding is not simple [1,2].

Overcrowding is due to the imbalance of the need for emergency care and the hospital’s availability to provide the service [3,4]. It is a problem not only for the emergency department (ED), but for the entire hospital [5,6].

In this perspective, the central role of the hospital, and not just of the ED, is underlined by the definition of the American College of Emergency Physicians, where overcrowding is defined as “a situation that occurs when the identified need for emergency services exceeds available resources for patient care in ED, hospital, or both” [7].

In agreement with this definition, the Australasian College for Emergency Medicine states in turn that, when the hospitalization capacity is no longer guaranteed by the inpatient wards, an imbalance is created between patient demand and the supply that should be guaranteed by the hospital system. This determines the overcrowding of the emergency department and the access block, which are configured as two indicators of the dysfunction of the hospital system itself [8].

According to another definition, overcrowding refers to the condition leading to the dysfunction of the emergency department due to the fact that the number of patients (awaiting visit, awaiting transfer, or undergoing diagnosis and treatment) exceeds either the physical or staffing capacity of the ED [9].

Overcrowding thus appears to be simply defined as the imbalance between the constant increase in healthcare demand and the lack of hospital beds, both in the context of individual departments and in the context of the ED [1]. Overcrowding is therefore intertwined with the factors capable of determining an obstacle in the correct functioning of an ED, including the number of patients awaiting visit, transfer, diagnosis, treatment, and, above all, hospitalization.

Despite different approaches and attempts using different parameters, there is no standard measure that can quantify crowding in a univocal and effective way [10,11].

It has been comprehensively demonstrated by various studies concerning the subject that hospital crowding also causes a delay in the diagnostic process and in the start of treatment, triggering a vicious circle that feeds the overcrowding itself [12,13,14,15].

In turn, overcrowding also has a negative impact on the triage process, with an increase in the number of patients who do not access triage, an increase in the triage time itself, and an increase in the length of stay (LOS) [16,17,18].

Several studies and meta-analyses have also observed that ED overcrowding is associated with an increasing trend of leaving the ED before undergoing medical examination and treatment [19].

In this narrative review, we would summarize the various issues to the phenomenon and some solutions to solve or decrease the workload in hospitals, because overcrowding can increase waiting times, delay in patient care, length of stay, morbidity and mortality, decrease quality of care, and patient satisfaction; it can determine negative effects at all levels of the urgent healthcare system [20,21].

## 2. Materials and Methods

In this narrative review article, we performed a computerized database search to identify relevant articles. We searched for published and ready-to-publish articles in bibliographic databases, including ISI Web of Science, PubMed, Science Direct, Scopus, Wiley online library, and Google Scholar.

In addition, the literature search also involved a manual search of bibliographies of the identified papers and relevant information to meet the objectives of this study. The keywords used in the search were the following: Overcrowding; Emergency Department; Length of Stay; Waiting time; inpatient boarding, Triage, Hospital Emergency Services, ED Patient Flow, Ambulance diversion, Emergency Outpatient Unit, Patient Safety The selection of these terms was made with the help of MeSH service in PubMed website databases and all non-English articles were removed.

## 3. Overcrowding: The Input–Throughput–Output Model

The causes of overcrowding can be classified into three categories: input, throughput, and output factors. These parameters are independent from each other, but they are interconnected and influenced by underlying contributors, making the phenomenon of overcrowding a multifactorial and complex one [2,22,23].

For a greater understanding of the crowding phenomenon, as well as to be able to consider means to quantify it and contain it, it is necessary to analyze the three aforementioned factors that contribute to its development [24].

The input–throughput–output model therefore appears useful for understanding the parameters that regulate the flow and capacity of the ED, but also represents a guideline for conceptualizing the same parameters in both the entire hospital setting and the health care system [23,24,25,26].

Input, throughput, and output factors can be defined as follows:

- Input factors: they are represented by factors determining patient access to the ED. They include the waiting time, the number of patients which arrived in the ED, as well as their severity and complexity. Input factors constitute one of the causes of crowding, but the least important [5,27,28,29,30,31]
- Throughput factors (internal factors): they are represented by the process time, meaning the time between taking charge of the patient and the outcome (diagnosis and decision: discharge, hospitalization, and transfer). They include all the complementary exams that are performed in the ED (laboratory analysis and imaging). These factors are also affected by the healthcare personnel (in terms of quality of work, shift work, burnout, drop in performance, respect for shifts, and holidays) [3,6].
- Output factors: they include patients boarding in the ED, availability of hospital beds, and the delay of transport (both internal and external) to leave the ED. The lack of hospital beds appears to be a fundamental cause of overcrowding, but so is the lack of home care. The reduction of beds (which in some realities have decreased by more than 50% in the last 20 years) is a worldwide phenomenon that has led to exit block, as well as to the collapse of the possibility of hospitalizing patients. Considering output factors, it is therefore evident that overcrowding is influenced by the fact that patients who should go to the ward are stationed in the emergency room and must continue to be assisted from a medical point of view [3,5,6].

Below, in the Table 1 is a summary of these factors and their characterization in detail [21]:

It is also necessary to consider that:

- ED cannot control input factors [32,33];
- High number of patients with non-critical issues is not a primary cause of overcrowding [2,22];
- *Boarding* has a great importance among the causative factors of overcrowding. Boarding is in fact capable of causing a considerable dissipation of resources, which are subtracted from new patients. These resources include space, beds, diagnostic imaging techniques, but also human resources, such as hospital staff. This generates an increase in LOS and negatively affects the output factors, perpetuating the maintenance of overcrowding [23,34,35] A great number of studies provide solutions to limit boarding, although this does not represent the only causative factor of overcrowding, but its resolution would seem mandatory to limit the phenomenon [21].
- Exit block has a strong impact on overcrowding and is directly connected with the output factors. The solutions that can be promoted to alleviate exit block; however, they must not affect the patients’ outcome [36].

All the issues analyzed so far can also negatively impact the workforce, making the emergency medicine ward less attractive from a career point of view. These parameters, including overcrowding, boarding, and block, would also be able to negatively affect the quality of learning of young doctors in training [37,38].

**Figure 1:**
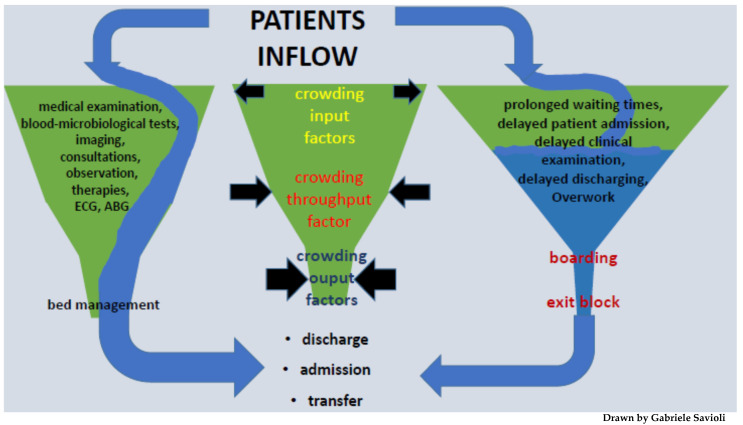
Diagram 1: Patient flow in emergency department.

Crowding can be effectively represented as a funnel: in the large part of the funnel are the catchment area and the input factors (number of patients, presentation methods); the process of ED patients takes place in the body of the funnel and therefore represents the throughput factors. The neck of the funnel instead represents the output factors. In the presence of crowding, it is as if the large part of the funnel is enlarged welcoming more patients while the body and the narrow part shrink, for example, due to increased process work (throughput factors) or due to lack of output factors, this causes stagnation and congestion in the flow of patients (see Figure 1).

## 4. Signs of Overcrowding

Signs of ED overcrowding include the following [9]:

- delay in the treatment of patients due to a lack of suitable spaces
- treatments administered in other spaces of the ED, including corridors
- prolonged stay of patients in the emergency room at the end of medical treatment, pending transfer to the ward
- inability to take care of patients transported by ambulance
- obstruction of the entry and exit routes of the ED.

## 5. Exit Block: Definition

The exit block occurs when “patients in the Emergency Department (ED) requiring inpatient care are unable to gain access to appropriate hospital beds within a reasonable time frame” [39].

The presence of exit block in turn determines a further aggravation of overcrowding, because a hospital already at maximum capacity will not be able to admit other patients [40]. In the presence of an exit block, patients are therefore bound to remain longer than necessary in the emergency department.

Exit block, as demonstrated by a major Australian study, causes an increase in the waiting time, and blockade can amount on average to up to 60% of transit time in the ED [41]. Exit block has important repercussions on several factors, including waiting times, boarding, impact on workforce, and, above all, patient outcomes. Several studies report how exit block is able to negatively influence the patient’s outcome, determining, for example, an increase in the waiting times of patients who must undergo surgery, even in an emergency regime. Likewise, an increase in waiting time and negative block rebound has also been shown in patients suffering from psychiatric diseases, especially in those requiring urgent treatment [40,42,43]. Moreover, exit block, just like overcrowding, could be one of the factors that lie behind a patient's decision to leave the ED before medical exam, resulting potentially in a worse outcome, depending on the pathology [44].

A series of studies have demonstrated the relationship that exists between exit blocks and shortage of beds: the shortage of hospital beds, associated with the reluctance and the slowness of the hospital wards to hospitalize patients, is certainly the major fundamental trigger of the exit block and long boarding, that is in turn a consequence of exit block [36,45]. In fact, in the days when the ED is overcrowded, an increase in both the LOS and exit block is observed [46].

In the same way, an increase in the occupancy of hospital beds determines the increase in overcrowding and exit block at the level of the ED [47]. Exit block therefore represents one of the main parameters used for the quantification of hospital dysfunction [42].

## 6. Boarding: Definition

Boarding has been defined as the practice of holding patients in the ED after they have been admitted to the hospital because no inpatient beds are available [48,49]. Boarding is therefore directly dependent on exit block.

One of the consequences of boarding is that the levels of assistance guaranteed by the staff, as well as the physical space, are exceeded [50], and this is because patients boarding in the ED require care usually provided by an inpatient care team [51]. It has been shown that in large Eds, 40% or more of staff time is spent caring for patients who have already been admitted to a hospital ward and are stationing in the ED while waiting for a bed, rather than looking after newly admitted patients [52].

Based on a single study, which analyzed waiting time in ED and the associated mortality, delayed hospitalization was negatively and independently correlated with increased mortality [53].

In another study, it was reported that the increase in boarding was not correlated with an increase in ED demand, reporting on the contrary a decrease in the number of patients and acute illnesses. However, it must be considered that the study reported an increase in the hospital admission rate while conducting the study [54].

In contrast to these results, an American study reported a reduction in boarding associated with an overall reduction in admission rate and LOS. Despite this, the same authors pointed out that the application of measures to counteract boarding (such as, for example, the movement of patients in corridors), could have contributed to misleading the analysis in the study [55].

## 7. The Access Block: Definition

According to the Australasian College for Emergency Medicine (ACEM) access block is defined as “the situation where patients are unable to gain access to appropriate hospital beds within a reasonable amount of time, no greater than 8 h” [56].

Access block also refers to the percentage of patients who were admitted or planned for admission but discharged from the emergency department (ED) without reaching an inpatient bed, transferred to another hospital for admission, or died in the ED, whose total ED time exceeded 8 h [9].

Access block for admitted patients that must remain in the ED awaiting a suitable inpatient beds and transfer to the ward is the principal cause of ED overcrowding [5].

Access block is mainly the result of hospital inpatient throughput and occupancy, and is therefore frequently beyond the direct control of the ED.

Access block has been linked to increased ED waiting time for medical care and therefore leads to ED overcrowding. It is in turn associated with increases in morbidity and mortality, and it is another factor that may lead patients to leaving the ED before receiving the essential treatment they need.

Access block currently represents a phenomenon of international significance and one of the main challenges of modern ED [56,57,58,59].

## 8. Overcrowding: Consequences

Despite the constant redefinitions of overcrowding and block, and the proposition of numerous solutions, the problem is far from being solved [60,61].

Overcrowding has been shown to have multiple consequences on several levels.

Overcrowding determines an increase in the risk and rate of adverse events, even serious ones, of morbidity and mortality, as well as an increase in the waiting time for definitive care. This relationship between overcrowding and mortality has been highlighted by several studies, both in the pediatric and adult populations.

Regarding the pediatric population, a Korean retrospective study showed that mortality at 30 days was higher in the pediatric population whenever they were admitted to an overcrowded ED [62].

Concerning the adult population, a Canadian cohort study demonstrated that the risk of death was increased by 34% at 10 days for patients who experienced ED overcrowding during hospitalization, compared to those who did not [63].

Finally, in an Australian retrospective stratified cohort analysis, it was found that in-hospital death within 10 days of presentation was higher in the population that had presented to the ED during an overcrowded shift [64].

Considering the impact of overcrowding on the increase of adverse events, an American retrospective cohort study demonstrated an increased rate of adverse cardiovascular events in patients with both acute coronary syndrome (ACS) related and non-ACS-related chest pain admitted to ED during overcrowded periods, compared to patients who did not experience overcrowding [14].

It has also been shown that overcrowding of the ED negatively affects the timing and modalities of the triage process as well, generating an increase in waiting times and LOS, with a consequent delay in the diagnosis and in the initiation of treatment. This in turn is responsible for a greater percentage of patients abandoning an overcrowded ED without being seen, as demonstrated by multiple studies [65,66,67,68,69].

Delayed initiation of treatment and prolonged waiting times (even among patients who should benefit from immediate care) lead in turn to patient dissatisfaction [16,70,71,72] and therefore negatively impact perceived waiting times, safety, and quality of care [64,73]. This was investigated in a prospective cross-sectional study, which included 644 patients, and highlighted an association between objective measures of ED crowding and perceptions of care compromise among patients and providers [74].

Another retrospective cohort study demonstrated that a poor ED experience as measured by waiting room times, ED boarding time after admission, ED treatment time, and location of the treatment and of the waiting time, are adversely associated with ED satisfaction and predict lower satisfaction with the entire hospitalization. Moreover, patients who went to the ED during a period of overcrowding were less likely to advise others to go to ED, in comparison to those who went to the ED during a period when overcrowding was absent [75].

It is eventually necessary to consider that overcrowding has a negative impact on the welfare of the medical personnel as well, as it represents one of the most important workplace related stressors [76].

## 9. Overcrowding and COVID Pandemic

In some recent works, we have shown that, in this pandemic, input factors played a modest/ambivalent role in crowding [3,6,74]. There are two main causes in ED crowding: output and throughput factors.

In terms of output factors, crowding was determined by the phenomenon of exit block, especially by the need for unprecedented care in medium- and high-intensity wards.

In a study conducted prior to this pandemic, through tabletop simulations of a potential maxi-emergency, our research group had anticipated that such a scenario was possible. In particular, we had shown how wards with high- and medium-intensity care could most easily determine boarding time and access block.

We believe this increment of access block is attributable to the discrepancy between the immediate and sudden need for intensive care (ICU) beds and the number of ICU beds available on the basis of national and local historical needs. However, it is important to emphasize that all patients, even those in need of low-intensity care, have struggled against access block. Therefore, the lack of beds seems to be the main cause of access block. Our opinion is that EDs are crowded when hospitals are crowded.

The waiting time for hospitalization was also prolonged because it was necessary to screen all patients before assigning them to a “clean” vs COVID-unit bed to ensure that infected (and perhaps asymptomatic) patients were not admitted to “clean” wards or wards in which the risk of infection had to remain low.

With regard to throughput factors, crowding has resulted from changes in the role of emergency physicians and EDs. Emergency departments are no longer merely where patients are sorted into specialist departments; patients are now treated and stabilized, and differential diagnostic tests.

## 10. Overcrowding: Possible Solutions

The resolution of overcrowding requires several actions, not only at the medical level, but also at the bureaucratic one.

One of the main possible solutions to the problem of overcrowding could be represented by an improvement in the access to care. Other potential solutions include an increase in transitional beds, and better working conditions (physical as well as psychological ones) for hospital staff [59,77].

As overcrowding is caused by a mismatch between supply and demand, one might think that an increase in supply (hospital beds and staff) could easily solve the problem. However, a problem that appears simple does not always have a simple solution: in some cases, EDs have increased their physical space and tried to improve the above parameters; however, this did not lead to an improvement in overcrowding and, on the contrary, a worsening of the situation was often observed [78]

Resolution strategies can be divided in two levels which act in synergy: micro- and macrolevel strategies [21].

### 10.1. Microlevel Strategies

Microlevel strategies can be applied in order to counteract the problem of overcrowding and boarding, and include those modifications that can be applied at the level of the Emergency Department [21].

The use of standardized diagnostic pathways, based on overt clinical pictures, can be extremely useful in the process of standardization of care, diagnosis, and treatment. They can reduce waiting times in specific subgroups, reducing the possibility of error and, in certain circumstances, hospitalization rates. They are also fundamental in improving outcome, reducing adverse events and mortality [1,79].

The coordination of integrated care within the ED, is another microlevel strategy that would help linking patients to alternative health care resources. On some occasions, patients access ED in the first instance as they are unable to navigate, due to various factors, within the healthcare system. This phenomenon is more widespread among certain social categories, such as low social classes, low literacy levels, as well as patients who experience fear of stigma and shame linked to certain conditions [80,81]. The reduction of overcrowding and access to EDs can be achieved through support from external or ambulatorial health services. Imaging techniques in non-critically ill patients could be devolved to other suitable facilities, in order to prioritize and ensure access to emergency diagnostic procedures for critical patients. Furthermore, the implementation of a follow-up system in a multidisciplinary perspective should allow a rigid and close monitoring of borderline patients who are discharged from the ED [82].

The setting of home care is another approach that, if well structured, can certainly play an important role in reducing ED overcrowding. Patients who do not require hospitalization can be discharged and continue home care after having been correctly diagnosed and subjected to stabilization and initial treatment in the ED.

The possibility of being able to continue treatment at home offers countless advantages to different populations of patients, especially the elderly, who find themselves in a familiar and comfortable environment, structured according to their needs, being able to continue care in a psychologically more congenial way [79].

In between the micro- and macrolevel strategies, is the institution of observation and short stay units. These units can help in reducing the number of patients that are stationing in the ED after diagnosis and initiation of treatment.

Patients who can benefit from the presence of an observation unit are those who, after diagnosis is made and treatment initiated, require a surveillance (for example after the introduction of a new treatment), or require complementary exams after a determined time interval, but that would not benefit from a prolonged hospitalization. Setting up short-stay units could therefore reduce ED overcrowding, allowing at the same time continuous patient monitoring and treatment [80].

An Italian research group has demonstrated that, in the years of activity of an OBI (osservazione breve intensiva, unit of short and intensive observation) Team, which had the function of flow coordination, stabilization of complex patients, boarding management, and bed management, a stabilization of boarding and exit block phenomena were observed, despite the increasing number of ED visits and the need for hospitalization of the patients themselves. A containment of LOS times and an improvement in the outcome of some categories of patients was observed as well [81,82,83,84,85,86,87,88]. These findings were in line with data from several other European and American research groups [89,90,91,92,93,94].

### 10.2. Macrolevel Strategies

Macrolevel strategies are solutions that can help counteracting the problem of overcrowding and boarding, and that have to be applied at the level of the hospital and/or the health-care system.

Among the macro-level strategies that can diminish crowding and boarding in ED, there are: simplification of the admission process, the establishment of a flow management center, intensification of the outpatient environment, integration of coordination and assistance that allow a more efficient transfer of patients within the hospital, redirection of elective and non-emergent work towards the outpatient setting, integration of the coordination of assistance and patient navigation programs within the hospital, better communication and connection amongst the hospital wards, and the development of hospital emergency plans. Commitment of hospital leadership and institutional awareness are fundamental at this level [21,22].

The simplification of the admission process and the flow management center would be able to guarantee a better control of the flow of patients, a better control of schedules for the transports, and would eventually allow a reduction of waiting times as well as an overall better organization of an overcrowded ED [95].

Another approach that can be used in order to diminish the overload of the ED, if applied systematically and wisely, is reverse triage. Reverse triage refers to the process of identifying hospitalized patients who are stable and do not require further treatment, and who can be discharged with no or little risk [96].

Reverse triage can be put in place through a targeted standardization of the treatment process, with the satisfaction of minimum criteria that would allow to discharge the patient.

Early hospital discharge is also facilitated and supported by proper collaboration with out-of-hospital structures, such as hospices, retirement homes, rehabilitation centers and the patient's home itself, alongside a correct support program, if necessary [96].

Reverse triage therefore constitutes another macrolevel strategy which can be used to rapidly create inpatient surge capacity, giving the priority to ED patients who require an urgent hospitalization, and in consequence diminish ED overcrowding.

The interventions described so far can be proposed, within certain limits, for the resolution of hospital overcrowding.

Awareness of the problem of overcrowding among the members of the hospital leadership is, however, a fundamental aspect that must be addressed in order to resolve the issue. The strategies outlined above need indeed to be structured, managed, and implemented at this level [97].

At last, if no improvement is seen, even despite the possibility of undergoing structural and organizational changes which could reduce the problem of overcrowding, enhanced regulations and legislations would be needed in order to regulate, through effective and precise guidelines, the problem of hospital overcrowding, pushing the problem and the need for resolution to a higher level [22].

## 11. Conclusions

Crowding is caused by input factors, throughput factors, and output factors. At the beginning, the input factors were the most studied but then they were found to be less relevant. The throughput factors have increased over time, switching from “admission to care” to “to care for admission”, and they are also affected by the number of staff and exhausting shifts. Currently, the block and boarding output factors are the most relevant and suffer from the exorbitant cuts in healthcare system over the world in recent decades.

As a result, the solution is to increase the number of hospital beds and medical and nursing staff. Instead, increasing the size of the ED was found to be counterproductive in some cases.

When the number of beds remains inadequate, solutions can be put in place to reduce or contain, but not solve, crowding.

These can be divided into micro- or macrolevel strategies.

Microlevel strategies can be applied in the emergency department, e.g., the use of standardized diagnostic paths and the establishment of a holding area.

Macrolevel strategies must be applied at the hospital and/or healthcare system level. Examples are the simplification of the hospitalization process, the establishment of a flow management center, the intensification of outpatient service, and the development of hospital emergency plans.

## Figures and Tables

**Table 1:** Factors contributing to overcrowding.

Parameter	Contributing Factors
Input	- emergencies (both medical and surgical) - visit type (both urgent and nonurgent) - ambulance arrivals - number of patients - triage score
Throughput	- time of processing - patients’ degree of gravity - process of triage and bed placement - bed availability (both in the ED and in the hospital) - staffing (nursing and other healthcare professionals), considering their experience and their training - other services (consultant and ancillary) - degree of boarding
Output	- hospital occupancy - inpatient bed shortage - transport delay (both internal and external) - staffing ratios - inefficient process of transferring care - inefficient planning of discharging patients - need of higher level of care - inpatients’ degree of gravity - lack of home care (both medical and not)

## Data Availability

Not applicable.
